# Emergency primary repair of grade V bladder neck injury complicating pelvic fracture

**DOI:** 10.1186/s13022-014-0004-8

**Published:** 2014-07-16

**Authors:** Elroy P Weledji, Pius Fokam, Djatche Nzade, Divine Eyongeta

**Affiliations:** 1 General & Visceral Surgeon, Regional Hospital Buea, Limbe, S.W. region, Cameroo; 2 Orthopaedic Surgeon, General Hospital Douala, Douala, Littoral region, Cameroon; 3 Radiologist, Regional Hospital Buea, Buea, S.W. region, Cameroon; 4 Urologist, Regional Hospital Limbe, Limbe, S.W. Region, Cameroon

**Keywords:** Pelvic injury, Bladder neck injury, Extravasation, Primary repair, Scrotal necrotizing fasciitis

## Abstract

We report a case of a grade V bladder injury complicating an open-book pelvic fracture following a road traffic accident. The bladder neck injury was primarily repaired in the emergency setting of a poor-resourced area with successful outcome. The dangers of urinary extravasation are still to be considered of importance and we advocate and encourage immediate/emergency open intervention although it remains controversial to say the least in a lesser resourced healthcare set up.

## Background

Pelvic ring disruption represents a high-energy injury with a high risk of life-threatening haemorrhage and damage to the bladder and posterior urethra. The risk of urethral injury is influenced by the number of broken pubic rami as well as involvement of the sacro-iliac joint [[1]]. Rupture of the posterior urethra is believed to be caused by shearing forces commonly occurring at the apex of the prostate. It is an uncommon but serious complication of anterior pelvic arch fracture. Immediate primary repair is not recommended in most cases of complete urethral disruption because the extensive haemorrhage, echymosis and swelling make division of planes and identification of anatomy and viable tissue extremely difficult [[2]]. It has been associated with higher rates of incontinence (25%), impotence (56%) and stricture rate (49%) [[1]] However, immediate exploration for repair is indicated for associated rectal injury, intraperitonel bladder rupture, bladder neck and prostatic urethral injury as they do not heal spontaneously and cause considerable morbidity including persistent and deep urinary extravasation [[3]].

## Case presentation

**Figure 1:**
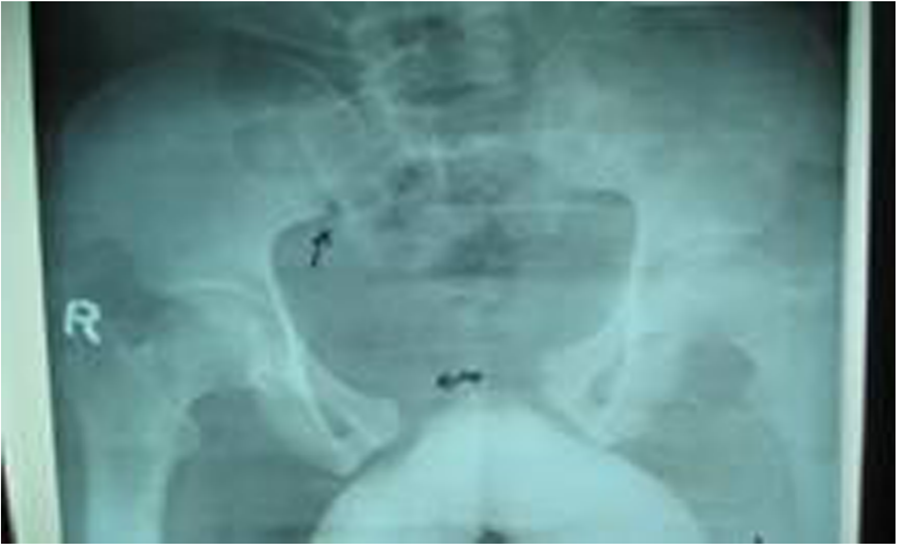
Open book pelvic fracture.

A 31- year old man was admitted as an emergency following a road-traffic accident in which as a pedestrian he was hit off the ground on the right side and fell from a height. He was conscious. The airway and cervical spine, chest and cardiovascular examinations were unremarkable. His blood pressure 112/59 mmHg, pulse 66/min, respiratory rate 26, and temperature 37°C. There were bruises over the lower abdomen and perineum and a progressive painful distension of the scrotum. There was a visible pelvic deformity with instability but no bleeding noticed from the external urethral meatus. A right upper leg deformity associated with bleeding from a small puncture wound was consistent with a compound fracture. This was immediately reduced, toileted and splinted. His pelvis was externally strapped to minimize movement. There was no distal neurovascular deficit. He was vigorously resuscitated with fluids and 2 units of blood and commenced intravenous antibiotics. Tetanus prophylaxis was administered. A chest x-ray was normal. An anterior-posterior (AP) pelvic film radiograph showed a Type B1, unstable antero-posterior compression fracture (open –book) with a separation of the pubic symphysis of about 8 cm (Figure 1). A radiograph of the limbs confirmed an unstable comminuted fracture of the proximal third of right tibia (Gustillo- Anderson Type II fracture) and dislocation of the proximal tibio-fibular joint. Gentle catheterization with a 12 F catheter by the attending physician failed. A digital rectal examination (DRE) revealed a high-riding prostate but no anorectal injury. The continuing enlarging scrotum was consistent with an extravasation of urine or an expanding haematoma. A percutaneous suprapubic catheter was inserted but drained just 6mls of fresh blood. The scrotal distension rapidly progressed to a tense imminent necrosis of the scrotal skin as the suprapubic drainage remained ineffective. Evacuation of the scrotum was avoided for fear of creating a urinary fistula. An open suprapubic cystostomy under local anaesthesia was attempted but abandoned because of the obscuring pelvic haematoma. A laparotomy was thus performed to control the persistent and deep urine extravasation.

**Figure 2:**
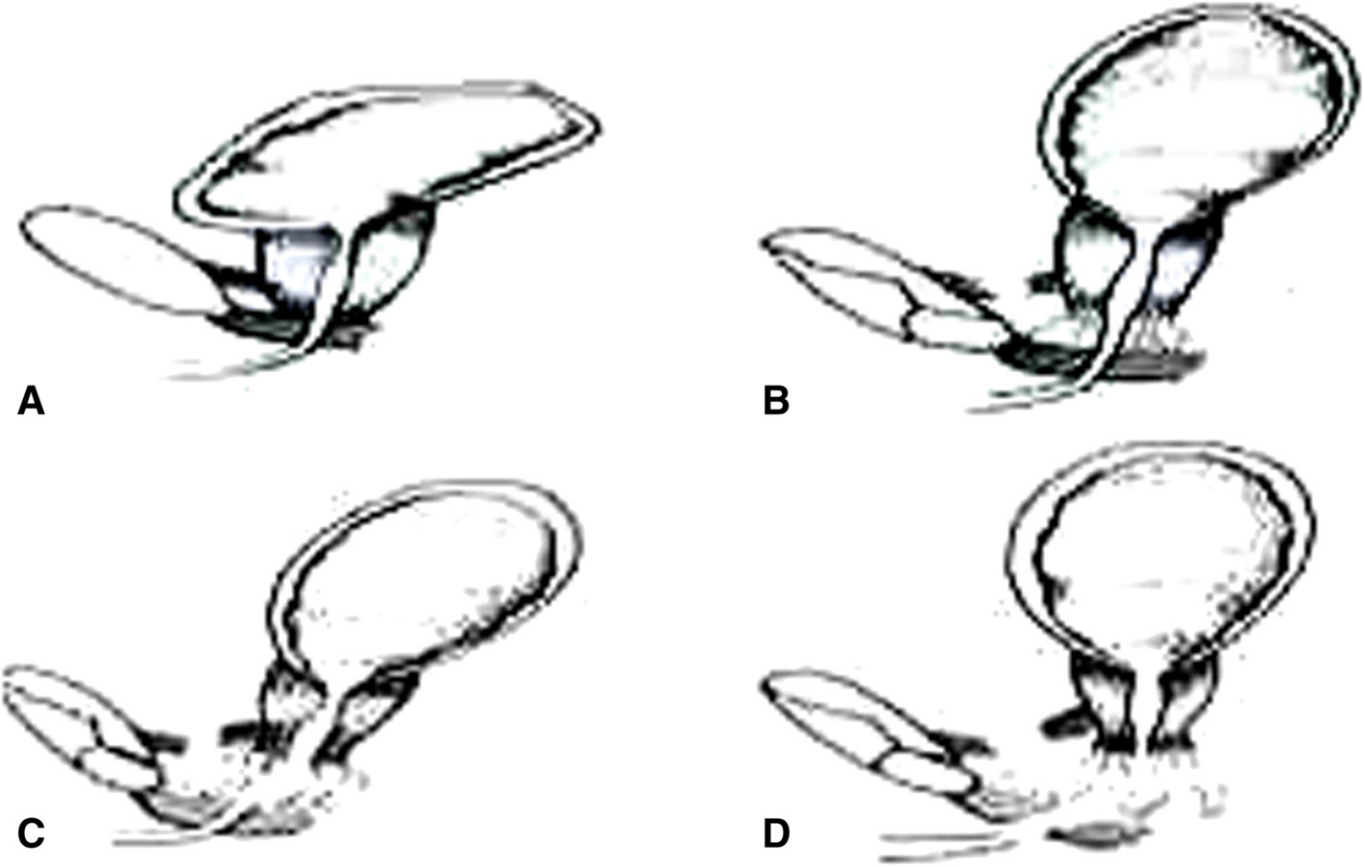
**Injuries of the urethra[**[7]**]. A**: The normal anatomy of the bladder, prostate, perineal membrane and puboprostatic ligaments. **B**: The pelvis is fractured and the puboprostatic ligament is ruptured but the urethra is only stretched and bruised. **C**: The pelvis is fractured and the puboprostatic ligament disrupted and there is complete tear through the membranous part of the urethra but the perineal ligament is intact. **D**: The pelvis is fractured, both puboprostatic and perineal ligaments are disrupted and there is a complete tear which involves the membranous and bulbous part of the urethra with an upward and backward dislocation of the prostate and bladder.

**Figure 3:**
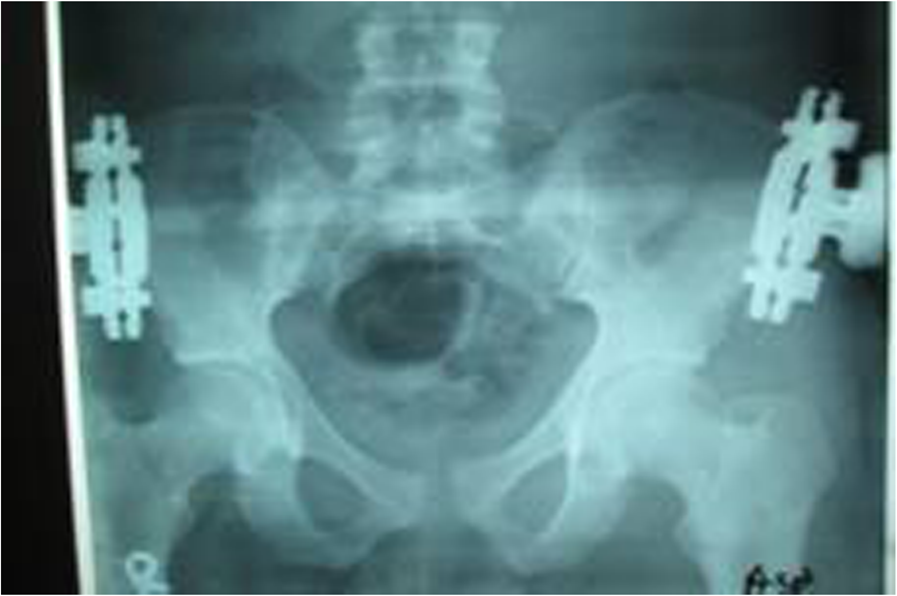
External fixation and reduction.

**Figure 4:**
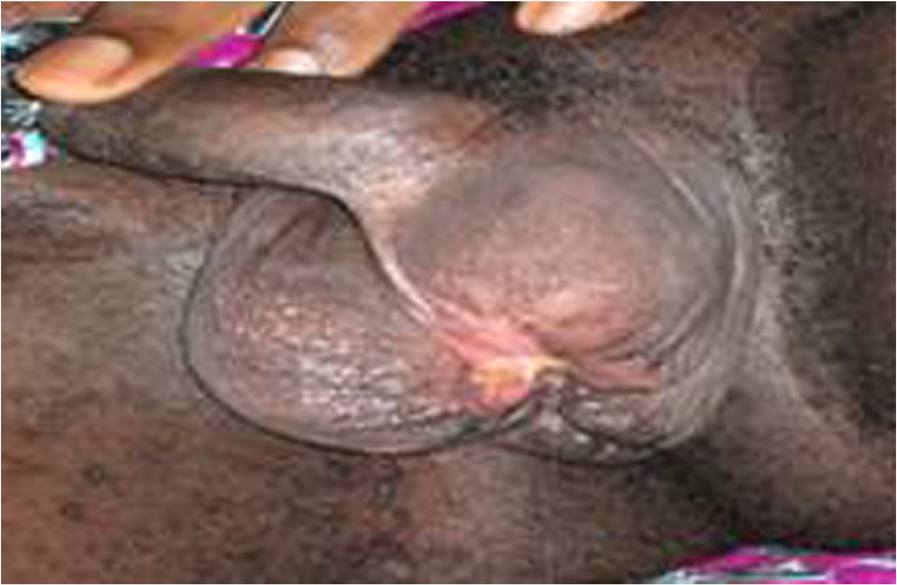
Scrotal wound: 2 months post excised necrotizing fasciitis complicating urinary extevasation- healing by second intention.

Following a low midline incision the prevesical and retropubic spaces were gently cleared - off blood (_~_ 5oomls) and urine that tracked extraperitoneally to the scrotum. The bladder neck was seen to be completely transected but did not extend to the trigone. It separated from the membranous urethra and was lying behind the bulb of the urethra by a wide gap of >5 cm. The perineal membrane was intact. Thus,a grade V bladder injury involving the prostatic urethra (Figure 2C, Tables 1 and 2). There was no other intra-abdominal injury. As there was no further bleeding and the patient was intraoperatively stable, a decision for primary repair was made to prevent this large amount of extraperitoneal extravasation of urine from the bladder neck. A *soft* 12 F catheter was gently passed retrogradely to the remnant of the posterior urethra and brought into the bladder neck. An all through continuous end –to- end anastomosis was fashioned with absorbable 2.0 vicryl over the indwelling catheter, beginning with the posterior layer and taking the puboprostatic ligaments on the sides. An interrupted anterior seromuscular layer completed the anastomosis. By applying traction to the catheter the balloon pulled the prostate down to the perineal membrane, thus opposing the cut ends of the urethra. A retropubic drain was inserted. A suprapubic catheter was thence properly fashioned as a cystostomy to decompress the anastomosis for a few weeks. The grade 1 compound fracture of the tibia was toileted and splinted with a cast. Post operative recovery was satisfactory. The following day the patient benefited from the insertion of an external fixator under local anaesthesia for stabilisation of the fracture dislocation of the pelvic ring and thereby protecting the anastomosis (Figure 3). However, a week later the patient developed a scrotal necrotizing fasciitis. This required an initial fasciectomy of the superficial perineal pouch enclosing the testes (Colles’ fascia) and thence recurrent debridement and toileting. He was put on a further two week course of intravenous ceftriaxone and metronidazole and the scrotal wound almost completely healed in a month (Figure 4). He had episodes of urinary tract infection with enterobacter sensitive to amoxycillin.

**Table 1:** **Bladder injury scale** [[12]]

Grade^*^	**Injury type**	**Description of injury**
**I**	**Haematoma**	Contusion, intramural haematoma
	**Laceration**	Partial thickness
**II**	**Laceration**	Extraperitoneal bladder wall laceration <2 cm
**III**	**Laceration**	**Extraperitoneal (≥2 cm) or intraperitoneal (<2 cm) bladder wall laceration**
**IV**	**Laceration**	**Intraperitoneal bladder wall laceration ≥2 cm**
**V**	**Laceration**	Intraperitoneal or extraperitoneal bladder wall laceration extending into the bladder neck or ureteral orifice (trigone)

Advance one grade for multiple lesions up to Grade III.

**Table 2:** **Urethral injury scale** [[12]]

Grade*	**Injury type**	**Description of injury**
**I**	Contusion	Blood at urethral meatus; urethrography normal
**II**	Stretch injury	Elongation of urethra without extravasation on urethrography
**III**	Partial disruption	Extravasation of urethrography contrast at injury site with visualization in the bladder
**IV**	Complete disruption	Extravasation of urethrography contrast and injury site without visualisation in the bladder, < 2 cm of urethra separation
**V**	Complete disruption	Complete transection with >2 cm urethral separation, or extension into the prostate or vagina

^*^Advance one grade for bilateral injuries up to grade III.

**Figure 5:**
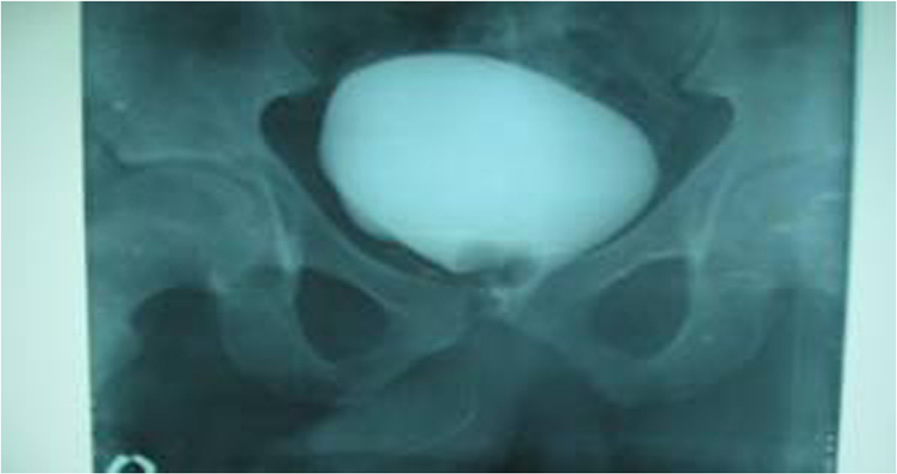
Retrograde cystogram: 2 months post posterior urethra repair.

**Figure 6:**
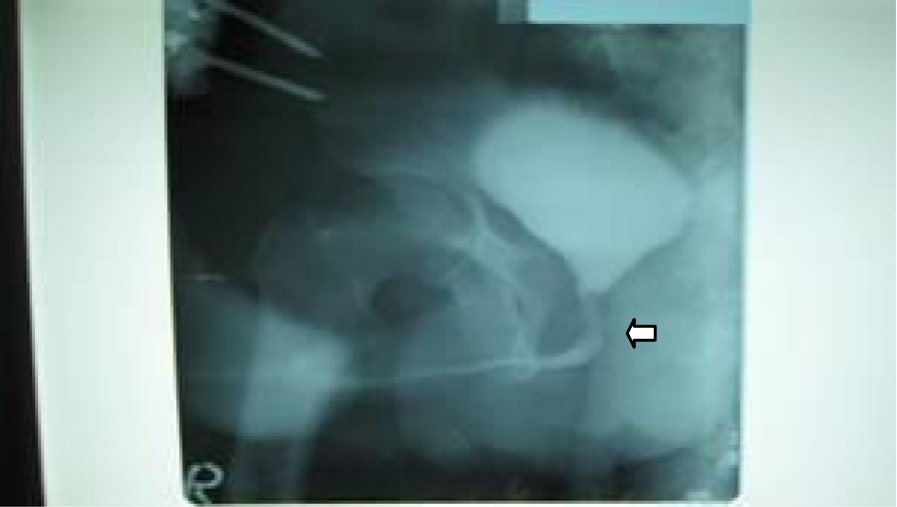
**Retrograde cystourethrogram:** ***arrow shows site of anastomosis*.**

2 months post urethral repair, a retrograde cystourethrogram revealed a completely healed anastomosis (Figures 5 and 6). The patient had a normal sensation of bladder-filling and normal voiding. He had no urge nor passive incontinence and, no erectile impotence as evidence of sphincteric or autonomic injury respectively. The external fixators were removed at this time following evidence of fracture healing and consolidation. The patient also benefited on this occasion with an internal fixation of his conservatively treated tibia fracture.

## Discussion

The case reports an uncommon emergency procedure or practice for a not so uncommon condition. It demonstrates the prompt treatment of a bladder neck transection after a pelvic fracture in order to decrease the considerable morbidity of deep extravasation of urine. The authors had gone to do an open cystotomy but then carried on to do a repair of the grade V bladder injury. This was not the primary intention as primary repair is not usually advisable even in resourced academic centres. Nonetheless, if conditions permit as in this case, where the fashioning of an effective suprapubic cystostomy was difficult, it is a good way of tackling the injury [[3]]. There is however a risk in some patients whose major remaining stabilizer is the musculature of the anterior abdominal wall as the tamponade effect on the pelvic haemorrhage may be lost with potential fatal consequences if iatrogenically disrupted by laparotomy [[4]]. The early management of an unstable pelvic fracture with haemodynamic instability is by external fixation carried out expeditiously as an emergency ‘resuscitation’ procedure [[5]]. By stabilizing the pelvis the haemorrhagic factors are counteracted [[4],[5]]. Correct reduction of the dislocated pelvis will also help bring the separated ends of the urethra nearly together and urethra injury repair will rely on the splinting effect of an indwelling catheter [[6]]. In this case the external fixator was used after the definitive repair, thus serving more for stabilization and protection of the repair than for resuscitation [[4]].

Following injury to the bladder neck, the internal sphincter goes into spasm and urine extravasates into the extraperitoneal spaces incuding the superficial perineal pouch enclosing the scrotum [[6]]. The disposition of Scarpa’s fascia of the anterior abdominal wall extending into the scrotum as Colle’s fascia, fusing laterally with fascia lata in the upper thigh and posteriorly with the perineal body to continue anteriorly as Buck’s fascia of the penis and then back up to the anterior abdominal wall prevents extravasation of urine into the thigh, abdomen and buttocks respectively. However, the extravasated urine must be prevented by successfully draining the bladder with a suprapubic catheter. Otherwise scrotal necrotizing fasciitis will rapidly occur as in this case (Figure 4). An evacuation of the scrotum may have prevented this complication. On hindsight the fear of creating a urethro-cutaneous fistula with its long-term complications was not founded as the prostatic urethra is far from the scrotum [[5]].

There was a high index of suspicion of urethral injury in this patient with the extravasation into the scrotum. A gentle urethrogram would ideally have been performed to image the injury in the emergency setting. Occasionally, when the general condition of the patient permits, an on- table cystourethrography is performed to fully reassess the extent of lower urinary tract injuries if the patient had been transferred promptly to the operating room [[7]]. Urethral tears are often complete because the urethra is damaged by the splintered rami. If the perineal membrane and the puboprostatic ligaments are also torn, the prostate and the neck of the bladder dislocates upwards and backwards and continuity of the urethra is completely lost (Figure 2D) [[6]]. This would make percutaneous bladder catheterization difficult as in this case. In the patient with a grade V urethral injury the perineal membrane is spared but there is complete transection of the posterior urethra with a wide urethral separation (Figure 2C). The timing of urethra injury repair is nearly always determined by the patient’s other injuries but the sooner this can be done the easier it may be as in this case [[4]–[7]]. Nonetheless, delayed primary repair and realignment after a few days to 2 weeks from time of injury has a theoretical benefit that pelvic haemorrhage has settled and unlikely to recur and patient is more stable [[8]].

None of the complications of urethral injuries (a) stricture, (b) impotence, (c) impaired ejaculation from bladder neck sympathetic denervation and (4) incontinence has so far occurred in this patient although long term follow-up is required [[5]]. The fact that the perineal membrane was intact may explain why the autonomic nerves were not damaged and continence not affected. Evacuation of the pelvic haematoma may have reduced tension on neurovascular bundles and the stretch effect on the urethra. Continence was also aided by the repair of the puboprostatic ligaments and the successful realignment of the urethra. Maintenance of potency indicates that there was no damage to the neurovascular bundle of the penis nor the pelvic autonomic nerves [[6]–[9]].

Of the three conventional treatment methods of posterior urethral injury, primary suturing of the disrupted urethral ends has the greatest complication rates of incontinence and impotence (56% and 21%). Primary realignment has doubled the incidence of impotence and half that of stricture compared to suprapubic cystostomy (SPC) and delayed repair (36% vs 19% and 53% vs 97%). The primary suturing approach is associated with the lowest stricture formation rate (49%) compared to initial SPC with delayed urethroplasty which has a stricture rate of 97%. Mouraviev et al. demonstrated that early realignment provided better outcomes and increased complications were not seen [[9]]. Immediate primary realignment of posterior disruption resulted in negligible intraoperative morbidity and acceptably low incidences of impotence, incontinence and symptomatic strictures on long term follow-up making it a worthwhile manoeuvre [[10]]. Further detailed analysis of studies have confirmed that primary urethral realignment is not associated with increased incidence of impotence and results in the formation of simpler strictures which can be managed by simpler techniques like urethrotomy and dilatation [[11],[12]]. This has reinforced the argument in favour of this approach.

## Conclusions

Primary emergency repair of bladder neck and posterior urethral injury complicating a pelvic fracture can be safe, feasible with minimal complications in the haemodynamically stable patient. The dangers of urinary extravasation are still to be considered of importance as primary repair is more effective and reassuring in preventing extravasation of urine than suprapubic cystostomy followed by delayed repair. An anterior frame external fixator would be required for stabilization of the pelvis and thus protect the repair. Communication is essential between orthopaedic and general surgeons when there is pelvic trauma.

## Consent

Written informed consent was obtained from the patients for publication of this case report and any accompanying images. A copy of the written consent is available for review by the Editor-in-chief of this journal.

## Competing interests

The authors declare that they have no competing interests.

## Authors’ contribution

PW is main author and visceral surgeon, PF is the orthopaedic surgeon, DN is the radiologist, DE gave some urological advice. All authors read and approved the final manuscript.

## References

[B1] Koraitim MM Pelvic fracture urethral injuries: the unresolved controversy J Urol 1999 161 1433 1441 10.1016/S0022-5347(05)68918-5 10210368

[B2] Sandler CM Harris JH Jr Corriere JR Jr Toouls BD Posterior urethral injuries after pelvic fractures Amer J Roentology 1981 137 1233 1237 10.2214/ajr.137.6.1233 6976098

[B3] Spirruck JP Pelvic fracture and injury to the lower urinary tract Surg Clin North Am 1988 68 1057 1069 3051452 10.1016/s0039-6109(16)44636-0

[B4] Botha A Brooks A Loosemore T Pelvic trauma Definitive Surgical Trauma Skills 2002 Royal College of Surgeons of England, London 85 88

[B5] Blandy J John B The Urethra Lecture notes on Urology 2005 5 216 235

[B6] Johnstone JMS Newham JE Rintoul RF Operations on the male urethra and genital organs Farquharson’s Textbook of Operative Surgery 1986 8 Churchill Livingstone, London 607 635

[B7] Kong JPL Bultitude M Royce P Gruen RL Cato A Corcoran NM Lower urinary tract injuries following blunt trauma: A review of contemporary management Rev Urol 2011 13 119 130 22114545 PMC3222924

[B8] Mundy AR Andrich DE Pelvic fracture-related injuries of the bladder neck and prostate: their nature, cause and management B J urol Int 2010 105 1302 1308 10.1111/j.1464-410X.2009.08970.x 19874306

[B9] Mouraviev VB Coburn M Santucci RA The treatment of posterior urethral disruption associated with pelvic fractures: comparative experience of early realignment versus delayed urethroplasty J Urol 2005 173 873 876 10.1097/01.ju.0000152145.33215.36 15711301

[B10] Elliot DS Barrett DM Long term follow up and evaluation of primary realignment of posterior urethral disruptions J Urol 1997 157 814 816 10.1016/S0022-5347(01)65051-1 9072573

[B11] Shrinivas RP Dubey D Primary urethral realignment should be the preferred option for the initial management of posterior urethral injuries Indian J. Urol 2010 26 2 310 313 20877620 10.4103/0970-1591.65416 PMC2938566

[B12] Moore EE Cogbell TH Jurkovich CJ Organ injury scaling.111: chest wall, abdominal, vascular, ureter, bladder and urethra J Trauma 1992 233 337 339 10.1097/00005373-199209000-00001 1404499

